# Exposure to High Precariousness Prevalence Negatively Impacts Drug Prescriptions of General Practitioners to Precarious and Non-Precarious Populations: A Retrospective Pharmaco-Epidemiological Study

**DOI:** 10.3390/ijerph19052962

**Published:** 2022-03-03

**Authors:** François Birault, Lakshmipriva Le Bonheur, Nicolas Langbour, Sandivanie Clodion, Nematollah Jaafari, Marie-Christine Perault-Pochat, Bérangère Thirioux

**Affiliations:** 1 Département de Médecine Générale, Faculté de Médecine et de Pharmacie, Université de Poitiers, F-86000 Poitiers, France; lak.lebonheur@gmail.com; 2 Département de Médecine Générale, Maison de Santé Pluriprofessionnelle Universitaire des Couronneries, F-86000 Poitiers, France; sclodion@hotmail.fr; 3 Centre Hospitalier Henri Laborit, Unité de Recherche Clinique Pierre Deniker, F-86021 Poitiers, France; nicolas.langbour@ch-poitiers.fr (N.L.); nemat.jaafari@ch-poitiers.fr (N.J.); berangere.thirioux@ch-poitiers.fr (B.T.); 4 Centre de Recherches sur la Cognition et l’Apprentissage, Centre National de la Recherche Scientifique (CNRS 7295), Université de Poitiers, F-86021 Poitiers, France; 5 Département de Psychiatrie, Faculté de Médecine et de Pharmacie, Université de Poitiers, F-86000 Poitiers, France; 6 Laboratoire de Neurosciences Expérimentales et Cliniques, Institut National de la Santé et de la Recherche Médicale (INSERM U 1084), Université de Poitiers, F-86000 Poitiers, France; marie.christine.perault.pochat@ch-poitiers.fr; 7 Service de Pharmacologie Clinique et Vigilances, Centre Hospitalo-Universitaire de Poitiers, F-86021 Poitiers, France; 8 Centre Hospitalo-Universitaire de Poitiers, Institut National de la Santé et de la Recherche Médicale (INSERM CIC1402), Université de Poitiers, F-86021 Poitiers, France

**Keywords:** precarious populations, reimbursed drug prescriptions, defined daily dose, primary care, treatment observance, medical efficiency, exhaustion, burnout, empathy

## Abstract

(1) Background: Precarious patients are more difficult to care for due to low literacy rates and poor adherence to treatment and hospitalization. These difficulties have detrimental effects on general practitioners (GPs), deteriorating medical communication, advice, diagnoses, and drug prescriptions. To better understand how precariousness affects primary care, we tested whether, among GPs, exposure to high precariousness prevalence more severely impacts drug prescriptions to precarious and non-precarious populations compared to low precariousness prevalence. Materials and methods: This pharmaco-epidemiological study, using linear regression analyses, compared the defined daily dose of 20 drugs prescribed by GPs to precarious and non-precarious patients in four French regions with low and high precariousness prevalence in 2015. (2) Findings: Exposure to high precariousness prevalence significantly impacted the prescriptions of nine medications to precarious patients and two medications to non-precarious patients, and distributed into three interaction patterns. (3) Interpretation: The selective over-prescription of drugs with easy intake modalities to precarious patients probably reflects GPs’ attempts to compensate for poor patient compliance. In contrast, the under-prescription of drugs targeting fungal infections in precarious populations and diabetes and cardiovascular diseases in non-precarious populations was seemingly due to a breakdown of empathy and professional exhaustion, causing medical neglect.

## 1. Introduction

Precariousness is a sociological construct [1] denoting a state of important material deprivation. It refers to the so-called “material need insecurities” [2], that is, poverty, unemployment, malnutrition, homelessness, lack of health insurance, and compromised access to education [2]. In 2015, precariousness affected about 13.5 % and 18% of the U.S. [3] and E.U. populations [4], respectively, and, within the E.U., up to 5.4 million individuals in France [5]. Precariousness rates remained stable worldwide from 2015 until the COVID-19 pandemic. However, estimates for the impact of the pandemic indicate a substantial increase in global precariousness. For years to come, up to 400 million new precarious people will live under the USD 1.90 poverty line as defined by the World Bank, and over 500 million under the poverty lines of USD 3.20 and USD 5.50 [6].

Precariousness is a significant predictor of poor health outcomes [2]. In fact, precarious populations present with specific medical characteristics, including a higher incidence of mental disorders, cancers, cardiovascular, neurological and metabolic diseases [7], and higher mortality [8,9]. For example, there is a risk factor of 1.4 for end-stage chronic kidney disease, cardiovascular or neurological disease, 2 for epilepsy, 2.2 for diabetes, 2.4 for psychiatric illnesses, and 4.7 for addictive disorders [10,11]. This also holds true for obesity (15.4% versus 9.0% in the general population) [10,12].

Consequently, because of their poorer health status, precarious people visit their general practitioners (GPs) more frequently [13]. Moreover, because of the higher incidence of specific chronic diseases in precarious patients, prescriptions of drugs targeting these diseases might be expected to be more frequently prescribed to precarious patients. However, this is not the case.

Recently, we carried out a large pharmaco-epidemiological study on the drug prescriptions rates of GPs across the entire French population [14]. We observed that GPs equally prescribe amoxicillin, pyostacine, ivermectin, salbutamol, and tiotropium to both precarious and non-precarious patients, although the former are more affected by diseases targeted by these medications (e.g., chronic bronchitis, bacterial pneumonia, cutaneous infection, etc.). Insulin glargine was found to be equally prescribed between populations, and metformin was under-prescribed, although diabetes is more prevalent in precarious populations [15].

A possible and systemic explanation for these findings is that the French healthcare system fails to compensate for selective inequalities in access to drug prescriptions. However, this cannot account for all the effects observed. Moreover, most French GPs intend to promote equal access to care in conformity with the ethical recommendations by the French healthcare system [16].

Another potential explanation is that the medical features specific to precariousness negatively impact the interaction between GPs and patients. In fact, precarious populations are more difficult to care for [17,18,19]. This is due to low rates of literacy and language competence as well as to significant comorbidities [20,21]. Because of their adverse socio-economic conditions, precarious patients have a greater tendency to neglect their own health status [21]. They also adhere less easily to treatment and hospitalization [21]. These difficulties probably lead GPs to adapt their prescriptions, seeking maximum efficiency, for example, by promoting drugs with easier intake modalities even if these are not recommended as first-line treatments. More seriously, these difficulties are also considered to exhaust GPs. Caring for precarious patients is well known to generate an extra workload, a considerable loss of energy, lack of positive feedback, and diminished personal accomplishment [21,22]. This dramatically impacts the prescription of drugs [14] as well as medical communication, advice, and, more importantly, diagnoses. Indeed, the quality of the patient–physician interaction varies depending on the patients’ characteristics [23,24], that is, age, gender, coping style, severity of conditions [24,25], education, social position, and ethnicity [24,26]. As an example, Bao et al. [26] reported a strong education and income gradient in the discussion of cancer screening strategies. That is, physicians are less likely to discuss the screening outcomes with their low-education and low-income patients. In the same vein, Kelly-Irving et al. [24] demonstrated that patients with a lower education level who evaluated themselves as having poor health were less reliably diagnosed as such by their GPs.

Taken together, these data indicate a negative effect of precarious features on the patient–physician relationship, significantly impacting the overall nature and quality of primary care [23,27,28,29]. However, to date, no pharmaco-epidemiological study has investigated whether daily exposure to high precariousness prevalence has more detrimental effects on GPs’ drug prescriptions compared to low precariousness prevalence.

Here, we tested whether, for GPs, there is an association between exposure to high precariousness prevalence and the subsequent inequalities of drug prescriptions between precarious and non-precarious populations. For this, we used a methodological approach based on the defined daily dose (DDD). This approach was already validated in our prior study [14]. In this previous work, the disparities observed in drug prescriptions could not be explained solely by epidemiological differences between populations and suggested a more general effect of exposure to precariousness on drug prescriptions. However, because our analyses were based on the entire French population, this potential effect of high precariousness prevalence was smoothed across regions. To overcome these limitations, we applied this DDD-based method to four French regions with different precariousness prevalence. We hypothesized that exposure to high precariousness prevalence, compared to low precariousness prevalence, more negatively and severely impacts GPs’ drug prescriptions to precarious patients, reinforcing inequalities between populations.

## 2. Materials and Methods

The present study is a quantitative, retrospective, and comparative study using a DDD-based methodological approach. We describe this method in detail below (see also [14]).

### 2.1. Precariousness Criteria in the French Healthcare System

According to the French healthcare system, people with a monthly income below EUR 720 for a single person and below EUR 1080 for a couple living in urban areas are considered precarious. In France, 89% of the population benefits from health care coverage. The French healthcare system reimburses both precarious and non-precarious people up to 65% of their health expenditure. Concerning the remaining 35%, non-precarious populations can sign up for additional private insurance, enabling them to be fully reimbursed (100%). In contrast, precarious people benefit from the support of the French healthcare system and are automatically reimbursed up to 100%. This “Universal Medical Coverage” (UMC), introduced in 2000, was substantially amended and its functioning significantly improved in 2015. Some 5.4 million individuals have benefitted from this measure (8.21% of the French population).

### 2.2. Data Source

Data were obtained from the “Institut Statistique des Professionnels Libéraux" (ISPL) (i.e., French Institute for Private Practitioners), which collects data from the “Système National d’Information Interrégimes de l’Assurance Maladie” (SNIIRAM)) (i.e., National Health Insurance Information System). SNIIRAM was established in 1999 at the request of French legislators who asked health insurance funds to systematically organize all the available data regarding public health in France. This was done in order “to more precisely determine and evaluate health care utilization and health care expenditure of beneficiaries” [30]. SNIIRAM gradually improved until 2016. SNIIRAM started to collate individual data from 2006 and its database was extensively developed in 2015 based on almost 66 million inhabitants and concerns—“prevalence and incidence of diseases, patient care pathways, health status and health care utilization of specific populations, real-life use of drugs, assessment of adverse effects of drugs or other health care procedures, monitoring of national health insurance expenditure, etc.” [30]. Moreover, “SNIIRAM comprises individual information on the sociodemographic and medical characteristics of beneficiaries and all hospital care and office medicine reimbursement, coded according to various systems” [30]. Here, we obtained individual data regarding reimbursed drug prescriptions and the health insurance status of each patient to whom these drugs have been prescribed. We were not allowed to obtain data concerning the patients’ age, gender, or underlying medical conditions. The data obtained are publicly available and anonymized. The study protocol and all methods were carried out in accordance with the relevant guidelines and regulations based on the permanent convention established between SNIIRAM and ISPL.

### 2.3. Data Selection

#### 2.3.1. Data Selection Regarding Prescribers, Populations of Patients, Year of Prescription, and French Regions

We extracted and compared the rates of private GPs’ drug prescriptions to precarious (P) and non-precarious populations (NP) in 2015 in four French regions with different precariousness prevalence.

**Prescribers**—We focused on GPs’ prescriptions for two main reasons. Firstly, GPs prescribe to the entire French population, regardless of gender. This is not the case for urologists, gynecologists, or midwives. Secondly, precarious populations have been well documented to visit their GPs more frequently than specialists [13]. Thirdly, primary care is the point of first contact between health systems and precarious people [30].

**Year of prescription**—We focused on the year 2015 because UMC was significantly amended and its functioning improved in 2015 (see above and [30]).

**Regions**—We focused on four French regions with different precariousness prevalence on the basis of the UMC annual statistics report. This was done to measure the impact of the precariousness gradient on GPs’ drug prescriptions.

Firstly, we selected two regions, that is, the Brittany region (BR) and the Center region (CR), with comparable insured populations and with low UMC populations. BR and CR have the two lowest insured populations in France.

− **BR**: 3,341,188 insured people, including 149,115 precarious individuals (4.46% of the regional population). We selected BR because it has the lowest UMC population compared to other French regions.
− **CR**: 2,635,080 insured people, including 172,758 precarious individuals (6.56% of the regional population).

Secondly, we selected two regions with different insured populations, similarly high UMC populations, and, consequently, different UMC prevalence—the Occitany region (Languedoc Roussillon and Midi Pyrenees) (OCR) and the Overseas region (Guadeloupe, Guyana, Martinique, and Reunion) (OSR).

− **OCR:** 5,771,360 insured people, including 515,259 precarious individuals (8.82% of the regional population).
− **OSR**: 1,890,901 insured people, including 613,453 precarious individuals (32.44% of the regional population). OSR has the highest UMC prevalence in France.

#### 2.3.2. Data Selection Relative to Medications and Criteria of Principal Evaluation

For medications, we used the Anatomical Therapeutic Chemical (ATC) classification. The World Health Organization (WHO [22]) Collaborating Centre for Drug Statistics Methodology is in charge of this classification. Medications are divided into different groups according to the organ or system on which they have an action, and according to their therapeutic and chemical features. This classification is based on five levels. The first level (first letter) indicates the anatomical group (among 14 different ones); the second level (first two numbers), the main pharmacological or therapeutic subgroups; the third and fourth levels (second and third letters), the therapeutic, pharmacological, or chemical subgroups; the fifth level (last two numbers), the chemical substance. This classification allows for a therapy to be characterized via analysis of the pharmaceutical used. As an example, metformin is a chemical substance corresponding to the fifth level (defined by the letters and numbers as follows: A10BA02) and belongs to the chemical group of biguanides (fourth level, A10BA) representing the pharmacological family of hypoglycemic agents (third level, A10B), a component of the therapeutic family of drugs indicated in diabetes (second level, A10), belonging to the anatomical group of the digestive system (first level, A) (Table 1).

We used the defined daily dose (DDD) as a criterion of principal evaluation. That is, we hypothesized that differences in reimbursed drug prescriptions are reflected in DDD variations for given medications between populations (same as in [14]).

Drug consumption can be expressed in cost, number of units, number of prescriptions, or by the physical quantity of drugs. The DDD is a technical unit of measurement and is determined by WHO. It refers to the assumed average maintenance dose per day for a drug used for its main indications in adults. DDDs are only assigned for medicines given with an ATC code. The DDD is noted on each pack by the producer and corresponds, thus, to a daily cost. Applying the DDD allows for monitoring changes in drug utilization over time, comparing and evaluating the effect of an intervention on drug use, documenting the relative therapy intensity with various groups of drugs, tracking changes in the use of a class of drugs, and evaluating regulatory effects and the effects of interventions on prescribing patterns [22].

Here, the expenditure rate was obtained based on the DDD per patient over 20 years of age for each pharmaceutical form and for each selected medication in 2015. For each ATC class, we selected two medications, depending upon two criteria—the medication with the highest total cost of reimbursement and the medication with the highest number of packs reimbursed. We used this methodology as some medications are sold at a high price, that is, in packs containing only low daily doses (i.e., for short-lasting treatments) (e.g., amoxicillin). In contrast, some medications are sold at lower prices, that is, in packs containing more daily doses (i.e., for long-lasting treatments) (e.g., atorvastatin). When a given medication for a given ATC class responded equally to these two criteria (total cost of reimbursement and total amount of packs reimbursed), this was uniquely retained. For the given ATC classes, there was, thus, only one medication selected. We note here that the antineoplastic class L was excluded, as GPs are not the prime prescribers for this class.

Similarly to our previous study, only one medication equally responded to the two selection criteria defined (i.e., total cost of reimbursement and total number of packs reimbursed) for four out of all first level ATC classes (the antineoplastic class L excepted). Thus, the same 20 medications as in our previous study were selected, that is, class A: metformin, insulin glargine; class B: acetylsalicylic acid, rivaroxaban; class C: atorvastatin, rosuvastatin; class D: econazole, ciclopirox; class G: *Serenoa repens*, tamsulosine; class H: prednisolone; class J: amoxicillin, pyostacine; class M: ibuprofen; class N: paracetamol; class P: ivermectin; class R: salbutamol, tiotropium; class S: cromolyn sodium, timolol.

### 2.4. Statistical Analyses

#### 2.4.1. Linear Regression Analyses

Statistical analyses were computed using Jamovi software © (open-source software, Jamovi Stats, Callaghan, Australia).

**Figure A1:**
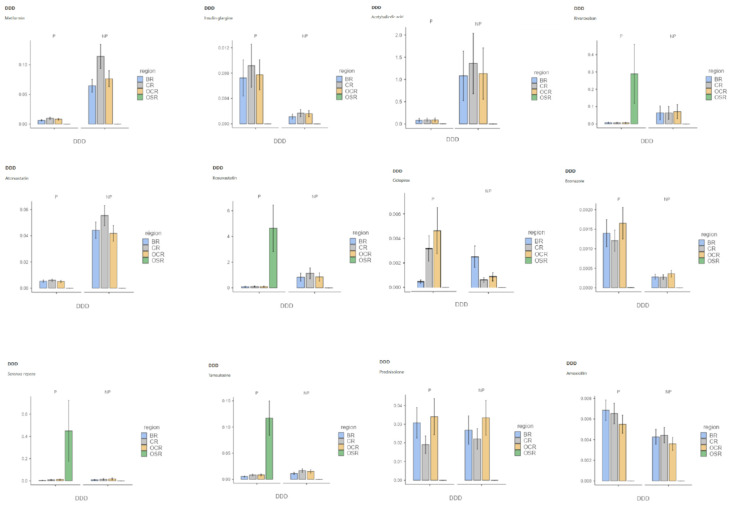

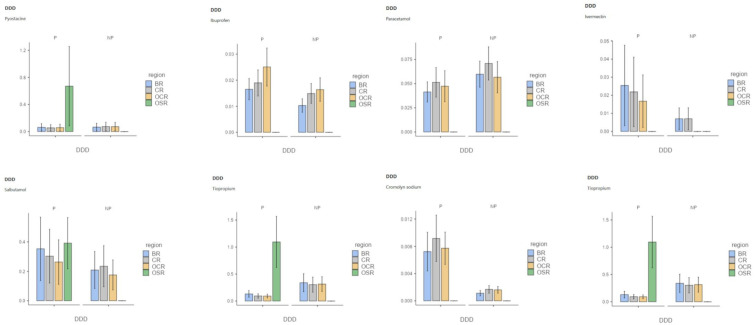
DDD distribution among precarious and non-precarious populations is shown for each tested molecule in each region (Note: DDD = defined daily doe; P = precarious populations; NP = non-precarious populations; BR = Brittany Region; CR = Centre Region; OCR = Occitany Region; OSR = Overseas Region).

We first calculated the number of reimbursed DDDs for a pack per precarious patient (P) and non-precarious patient (NP) in each region. Secondly, we computed the mean DDD for each medication and for all packs in each region (Table 1; for more details on the DDD distribution among the P and NP for each medication in each region split into graphs, see Appendix A Figure A1).

To test whether exposure to high precariousness prevalence impacted the GPs’ drug prescriptions to precarious populations compared to non-precarious populations, we calculated linear regression with the DDD values as dependent variable, the rates (in percentage) of precariousness prevalence for each region as covariates, and the populations (precarious (P); non-precarious (NP)) as factors (confidence interval: 95%; population effect: *t*-test; prevalence effect: *t*-test; population * prevalence interaction: *F*-test). This was done for each of the 20 selected medications (for details on the assumption verifications prior to the linear regression analyses, see Appendix B Table A1).

#### 2.4.2. Required Sample Size and Achieved Power Computations

We computed (1) the DDD sample size on the basis of the R^2^ obtained in the linear regression analyses for each tested medication and (2) the achieved power on the basis of the sample number for each tested medication (Table 2) (α err. prob. = 0.005; power (1-β err. prob.) = 0.8; number of predictors = 3). Because the achieved power for ivermectin and salbutamol was found to be <0.8, these two medications were removed from the statistical testing.

## 3. Results

### 3.1. Effect of Populations on GPs Drug Prescriptions

DDD significantly differed between P and NP for 12 out of the 20 selected medications, that is, metformin, insulin glargine, acetylsalicylic acid, rivaroxaban, atorvastatin, rosuvastatin, econazole, tamsulosine, amoxicillin, tiotropium, cromolyn sodium, and timolol. The mean DDD was significantly higher in the P than the NP for 9 out of the 12 medications, including amoxicillin, cromolyn sodium, econazole, rivaroxaban, rosuvastatin, tamsulosine, timolol, and tiotropium. In contrast, the mean DDD was significantly lower in P than NP for metformin, acetylsalicylic acid, and atorvastatin (Table 3).

There was a significant positive effect of the precariousness prevalence on drug prescriptions. That is, the more the precariousness prevalence increased, the more the DDD increased for eight medications: metformin, acetylsalicylic acid, rivaroxaban, atorvastatin, prednisolone, amoxicillin, ibuprofen, and paracetamol (Table 3). The inverse pattern (i.e., a DDD decrease) was not observed.

### 3.2. Effect of the Interaction between Precariousness Prevalence and Population on GPs’ Drug Prescriptions

There was a significant effect of the interaction between precariousness prevalence and population on the DDD for 11 medications, that is, metformin, rivaroxaban, atorvastatin, rosuvastatin, econazole, *Serenoa repens*, tamsulosine, pyostacine, tiotropium, cromolyn sodium, and timolol (Table 3).

**Figure 1:**
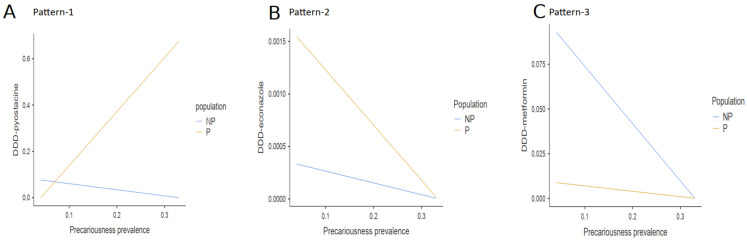
Interaction effects between precariousness prevalence and population on the DDD. There was a significant interaction effect between precariousness prevalence and population on the DDD, distributed into three different patterns. (**A**) In pattern-1, the more the precariousness prevalence increased, the more the DDD increased for rivaroxaban, rosuvastatin, Serenoa repens, tamsulosin, pyostacine, tiotropium, and timolol selectively in precarious populations. The DDD slightly decreased in non-precarious populations (data are shown for pyostacine). (**B**) In pattern-2, the more the precariousness prevalence increased, the more the DDD decreased for econazole and cromolyn sodium in precarious populations (data are shown for econazole). (**C**) In pattern-3, the more the precariousness prevalence increased, the more the DDD decreased for metformin and atorvastatin in both non-precarious populations (data are shown for metformin) (Note: NP = non-precarious; P = precarious; DDD = defined daily dose).

Significant interactions were distributed into three different patterns. In the first pattern (pattern-1), the more the precariousness prevalence increased, the more the DDD increased for rivaroxaban, rosuvastatin, *Serenoa repens*, tamsulosine, pyostacine, tiotropium, and timolol in precarious populations, selectively (Table 3; Figure 1A).

In the second pattern (pattern-2), the more the precariousness prevalence increased, the more the DDD decreased for econazole and cromolyn sodium in precarious populations selectively. There was also a trend for amoxicillin (Table 3; Figure 1B).

In the third pattern (pattern-3), the more the precariousness prevalence increased, the more the DDD decreased for metformin and atorvastatin in non-precarious populations selectively (Table 3; Figure 1C).

## 4. Discussion

In the present pharmaco-epidemiological study, we investigated whether, among GPs, there is an association between the exposure to high precariousness prevalence and the subsequent inequalities of drug prescriptions between precarious and non-precarious populations. More precisely, we tested whether exposure to high precariousness prevalence more negatively and severely impacts GPs’ drug prescriptions to precarious populations compared to exposure to low precariousness prevalence. For this, using a DDD-based methodological approach and linear regression analyses, we compared GPs’ prescriptions of 20 given medications to precarious and non-precarious populations in four French regions with respectively low (BR, CR) and high (OCR, OSR) precariousness prevalence.

In line with our working hypothesis, we demonstrated that the interaction between precariousness prevalence and population significantly impacted the prescriptions of 11 out of the 20 tested molecules. This was the case for metformin, rivaroxaban, atorvastatin, rosuvastatin, econazole, *Serenoa repens*, tamsulosine, pyostacine, tiotropium, cromolyn sodium, and timolol. These interactions were distributed into three different patterns, depending upon the diseases these medications target and the populations. Partially contradicting our initial hypothesis, this negative effect of exposure to high precariousness prevalence affected both precarious and non-precarious populations, although the former were more significantly impacted.

The first interaction pattern was the most prevalent. It concerned 7 out of the 11 listed medications, including rivaroxaban, rosuvastatin, *Serenoa repens*, tamsulosine, pyostacine, tiotropium, and timolol. The features of the first pattern are as follows. Firstly, these medications were slightly less prescribed to precarious populations when GPs were exposed to the lowest rate of precariousness prevalence. In fact, these should have been either equally prescribed between the populations (i.e., rivaroxaban, *Serenoa repens*, tamsulosine, timolol) or over-prescribed in precarious populations (i.e., rosuvastatin, pyostacine, tiotropium). This first finding suggests that exposure to low precariousness prevalence reinforces inequalities in drug prescriptions for precarious patients. It means that GPs, when they are not accustomed to precariousness, tend to care for precarious patients as for the general population, that is, without taking into account their clinical characteristics and epidemiological specificities [24]. Secondly, the more the precariousness prevalence increased, the more these seven medications were prescribed to precarious populations, selectively. Thirdly, these medications were over-prescribed to precarious patients when GPs were exposed to the highest rate of precariousness prevalence (Figure 1A).

We firstly discuss the increase and over-prescription of *Serenoa repens*, tamsulosine, and timolol in precarious populations within the first pattern. *Serenoa repens* and tamsulosine are alpha-blockers (G class) and target benign prostate hypertrophy (BPH) in men, aiming to reduce associated low urinary tract symptoms (LUTS) [31]. Timolol, a beta-blocker, is indicated for open-angle glaucoma [32]. LUTS and open-angle glaucoma are known to more frequently affect older men and older people, respectively. However, in France, there are more female than male UMC beneficiaries. Indeed, although the percentages of women and men are equally distributed in the 20–60-year bracket of the French general population, female UMC beneficiaries account for 58% of the 20–40-year bracket, 54% of the 40–60-year bracket, and 53% of the over-60-year-olds [5]. Furthermore, UMC beneficiaries are younger than the general population; 44% are under 20 years of age and half are adults from 20 to 59 [5]. This significantly contrasts with the general population, which is equally distributed across the four standard age brackets. Accordingly, because UMC beneficiaries are mostly young women, prescriptions of *Serenoa repens*, tamsulosine and timolol in our study should have decreased simultaneously with the increase in precariousness prevalence. This should have been especially observed in the OSR, which has the highest rate of precariousness prevalence across the four French regions tested. This was not the case.

Moreover, regarding LUTS and ethnicity, recent epidemiological findings suggested that the frequency [33,34,35,36] and severity of the disease [37] are significantly higher in Black African people. These findings are consistent with the over-prescription of *Serenoa repens* and tamsulosine in the OSR as 35% to 75% of OSR populations are Black African descendants (e.g., Guyane: 40%; Martinique: 75%; Réunion: 35%). However, there is a lack of consensus concerning the potential association between LUTS and precariousness. Indeed, the risk factor for developing LUTS has been found to not differ between precarious and non-precarious populations according to both French and international studies [8,37]. However, Kuo et al. [38] reported opposite results. Thus, the over-prescription of *Serenoa repens*, and tamsulosine in our data probably reflects ethnic risk factors for LUTS. There is also a potential effect of precariousness, although this explanation needs to be taken with caution.

The risk factor for open-angle glaucoma has been found to correlate with ethnicity but not with precariousness [39]. Even if partly in line with our results, these findings cannot explain why the over-prescription of timolol selectively targeted precarious populations in the OSR, nor why this over-prescription was also observed in the OCR, where most of the population is Caucasian.

There is also a second plausible explanation for these increased prescriptions of *Serenoa Repens*, tamsulosine, and timolol that is not based upon ethnic or more general epidemiological factors. In fact, a promising alternative to pharmacological treatments for both BPH and open-angle glaucoma is laser surgery [32]. Precarious people cannot afford these additional costs. Consequently, when exposed to high precariousness prevalence, GPs cannot mobilize the financial resources needed to compensate these additional costs, probably leading them to over-prescribe *Serenoa repens*, tamsulosine, and timolol. Therefore, we postulate that the over-prescription of these three medications reflects a failure of the French healthcare system to compensate for inequalities in access to care. This cannot be considered a deleterious effect of exposure to high precariousness prevalence, contradicting our working hypothesis.

As mentioned above, the prescriptions of rivaroxaban, rosuvastatin, tiotropium, and pyostacine also significantly increased with the precariousness prevalence. These findings conform to epidemiological factors. Indeed, the incidence of the pathologies these medications are indicated for is higher in precarious populations, that is, cardiovascular pathologies, hypercholesterolemia, severe asthma and bronchopulmonary diseases, and bacterial infections, respectively. Thus, the positive correlation between the DDD increase for these specific medications and the precariousness prevalence potentially indicates that exposure to high precariousness prevalence positively impacts the way GPs care for cardiovascular, metabolic, bronchopulmonary, and bacterial infections in precarious patients. This suggests, thus, that GPs improve their clinical competencies when exposed to high precariousness on a daily basis. They learn how to more efficiently care for precarious patients according to their medical features and adapt their clinical practice to their patients, rebalancing the inequalities between populations. This partly contradicts our hypothesis.

However, a second potential explanation is to be found in the galenic forms of these four drugs. Rivaroxaban and rosuvastatin are characterized by easy intake modalities, that is, once a day. Tiotropium, which is always used in association with other medications and is indicated for severe asthma and bronchopulmonary diseases, is also administrated in a single daily dose. According to the current recommendations regarding antibiotics prescriptions, pyostacine should not be prescribed as a first-line but only a second-line treatment. However, this medication has a short-duration prescription, a high level of efficiency, a low incidence of allergic reactions and antibiotic resistance [40], and, again, is administered once a day. Pyostacine is, thus, much easier to prescribe than amoxicillin. Therefore, these increased prescriptions of rivaroxaban, rosuvastatin, tiotropium, and pyostacine do not only reflect epidemiological factors but also the goal of efficiency. Indeed, GPs can more efficiently provide precarious patients, who generally present with low rates of literacy and language competence [41], with adapted advice regarding treatments with easy intake modalities. This probably leads to improved compliance in patients. However, it cannot be entirely ruled out that GPs choose to preferentially prescribe treatments with simple intake modalities in order to significantly diminish the time, energy, and mental investment needed to explain treatment modalities and self-administration and, thus, to prevent their own exhaustion. Therefore, the choice of drugs with easy galenical forms and their associated over-prescriptions would potentially reflect protective mechanisms and self-oriented behaviors in GPs. However, the present study does not enable us to disentangle whether this increased prescription is due to the specific epidemiological factors of precariousness or to antagonistic motivations in GPs, that is, a quest of efficiency vs. protective mechanisms.

The second interaction pattern concerned the prescriptions of econazole and cromolyn sodium. Econazole targets dermatological infections such as mycoses. Cromolyn sodium, in France, is prescribed to treat allergies. The features of the second pattern are as follows. Firstly, these two medications were over-prescribed to precarious populations when GPs were exposed to the lowest rates of precariousness prevalence in comparison to non-precarious populations. This effect was expected. Indeed, fungal infections have a higher incidence in precarious populations, as poverty fosters inadequate hygiene and unfavorable working conditions [42]. This first result suggests that GPs, when treating fungal and dermatological diseases, appropriately adapt to the clinical specificities of precarious populations, as opposed to the first pattern, even if they are exposed to low precariousness prevalence and are, thus, less accustomed to precariousness. Secondly, the more the precariousness prevalence increased, the less cromolyn sodium and econazole were prescribed. Although observed in both populations, this decrease in the prescriptions was much more significant in precarious populations. Thirdly, these two medications were comparably prescribed between populations when GPs were exposed to the highest rate of precariousness prevalence (Figure 1B). Hence, for dermatological infections, exposure to high precariousness prevalence reinforces inequalities between populations and severely impacts precarious patients.

Four main hypotheses can be given to explain these findings. Firstly, fungal diseases are difficult to correctly diagnose and there is, thus, a general lack of medical training regarding these diseases in primary care. As an example, GPs do not diagnose mycoses as easily as other diseases and do not know how to efficiently care for patients with these afflictions [43], leading them to under-prescribe adapted antifungal treatments. Because precarious populations suffer more frequently from fungal infections, they are more negatively impacted by this lack of training. Secondly, antifungal treatments need to be maintained for a period of six months, with one or two topical applications per day. As mentioned above, treatment compliance has been shown to be significantly weaker in precarious populations [21]. This probably leads GPs to under-prescribe antifungal drugs, anticipating that these treatments will not be observed appropriately. Thirdly, there are significant risks of interaction between econazole and most medications because of the cytochrome P450 inhibition, which could also have an effect on prescriptions. A fourth explanation for these results is that exposure to high precariousness prevalence, as stated in the introduction, causes an extra workload and an increased mental investment, exhausting GPs [21]. On the basis of a previous neuro-phenomenological work [44], we postulate that exhausting working conditions impair GPs’ clinical empathy, which is considered a non-negligible risk factor for burnout in medical care [44,45]. Indeed, it has been shown that GPs, when aiming to both alleviate distress and control emotional exhaustion (the first burnout symptom) within the care relationship, develop a coping strategy in which they distance themselves mentally from their patients [46]. This so-called “cynicism” or “depersonalization” is the second clinical symptom of burnout in physicians. It consists of negative, distant, and/or impersonal attitudes towards patients, leading physicians to mentally reject their patients [46]. These two sequential symptoms of burnout have a deleterious effect on the quality of care, with detrimental repercussions on patients’ health, and are associated with an increase in negligence and medical errors [47,48]. Hence, we assume that repeated exposure to difficult working conditions and medical situations encountered with precarious populations and repeated experiences of failures in care efficiency trigger a complete breakdown of clinical empathy, causing burnout syndrome. Thus, in the present case, under-prescriptions of econazole and cromolyn sodium potentially reflect GPs’ neglect of fungal and allergic diseases that they further considered, although wrongly, benign afflictions, due to exhaustion and potential burnout syndrome. This is in accordance with a recent cross-sectional descriptive study by Yugero et al. [49] based on 108 GPs and 183,600 patients in Spain, showing that GPs who are older, more empathic, and present less severe burnout symptoms had better prescribing performances. This was the case, for example, for diuretics and hypertension treatments. This association between altered empathy, burnout, and weaker prescription performance needs to be tested in future studies, as the same group also reported that sick leave prescription in GPs is not associated with empathy and burnout [50], contradicting the results quoted above.

The third interaction pattern concerned prescriptions of metformin and atorvastatin. These two medications are indicated for diabetes, and hypercholesterolemia and cardiovascular diseases, respectively. The features of the third pattern are as follows. Firstly, metformin and atorvastatin were over-prescribed to non-precarious populations when GPs were exposed to the lowest rate of precariousness prevalence. However, these drugs should have been found to be prescribed more in precarious patients, as diabetes and cardiovascular diseases have a higher incidence in these populations [51,52]. Again, this effect suggests that exposure to low precariousness prevalence reinforces the inequalities in drug prescriptions. This finding also indicates that GPs, when not accustomed to precariousness, do not appropriately care for diseases that more frequently affect precarious populations. Secondly, the more the precariousness prevalence increased, the less metformin and atorvastatin were prescribed to non-precarious populations, selectively. Thirdly, these were comparably prescribed between populations when GPs were exposed to the highest rate of precariousness prevalence (Figure 1C). Therefore, in the third pattern, as in the second, exposure to high precariousness prevalence negatively impacted GPs’ prescriptions. However, these two patterns are distinct in that under-prescriptions selectively concern precarious patients in the second pattern but non-precarious patients in the third pattern. Moreover, they concern fungal infections in the second pattern but the two leading causes of death worldwide in the third pattern [53]. This was unexpected. There is no suitable epidemiological explanation for this finding. This further contradicts studies reporting a strong education and income gradient in the physician–patient relationship and, thus, the quality of care [26]. Again, one plausible explanation is that this under-prescription reflected exhaustion and associated risks of burnout in GPs. Therefore, these results suggest that exhaustion, generated by the difficulties in caring for precarious patients, also had a significant impact on how GPs care for non-precarious patients. This double dissociation between more benign diseases (fungal infections) vs. more serious diseases (diabetes, cardiovascular pathologies) and precarious vs. non-precarious patients needs to be more deeply investigated in future studies.

The prescriptions of acetylsalicylic acid, ibuprofen, paracetamol, ciclopirox, prednisolone, and insulin glargine were not found to be impacted by precariousness prevalence. The first three drugs correspond to over-the-counter (OTC) analgesics and anti-inflammatory medications in France. This is also the case for ciclopirox, targeting onychomycoses. As such, these OTC drugs do not necessitate prescriptions. This may explain why no significant effect was observed. Prednisolone has multiple indications, principally cancers, rheumatisms, and infectious and pulmonary-allergy diseases, and thus, does not target pathologies that are more specific to precarious populations. This also may account for the absence of effect of precariousness prevalence in the present work. In contrast, our findings regarding prescriptions of insulin glargine are unexpected and more difficult to interpret. Replication studies are needed to better understand this absence of effect.

Overall, our study demonstrates that GPs’ daily exposure to high precariousness prevalence negatively impacts drug prescriptions and, thus, the quality of care. This effect concerns both precarious and non-precarious populations and probably originates in a complete breakdown of empathy and associated burnout syndrome. Consequently, future studies need to be carried out to test for the association between prescriptions modulations in GPs, precariousness prevalence, empathy breakdown (using the Interpersonal Reactive Index (IRI) [54]), and burnout symptoms (using the Malash Burnout Inventory (MBI) [46]). This would enable (a) the determination of whether the deterioration of empathy is associated with the onset of burnout in GPs who are exposed to high precariousness prevalence, (b) an improved understanding of whether this co-occurrence of disorders is a predictive factor of inadequate drug prescriptions, and (c) the early detection of red flags indicating the onset of medical neglect in GPs’ medical practice. Secondly, although constant exposure to precarious patients is considered to exhaust GPs, there is, paradoxically, no pedagogic tool teaching GPs how to adapt their practice to this population, that is, how to better take into account the specific medical features of these patients, to preserve their empathic skills, and to protect themselves from emotional exhaustion and depersonalization. Developing new training programs that teach both medical students in their initial medical training and GPs in continuous professional formations how to develop and preserve their clinical empathy and diagnose their own risk for burnout is an important new axis of research.

There are two main limitations to the present study. Firstly, we did not obtain data relative to the patients’ age, gender, health status, underlying medical conditions, risk factors, results of clinical examinations, reasons for or diagnosis or medical or paramedical consultations, results of laboratory tests, histology, pathology, drugs delivered in hospital, whether or not the drugs were taken, the prescribed dosage of drugs, and so on (for more details, see [8]). Consequently, we conducted neither a weighting nor stratification test. This may have led to significant confusion biases. Secondly, we focused our analyses on the numbers of reimbursed drug prescriptions. This means that it was not possible to estimate the proportion of irreducible inequalities, that is, the proportions of precarious patients who do not access care, as GPs refuse to provide consultation to these populations. This may have introduced a non-negligible selection bias.

## 5. Conclusions

The aim of the present retrospective pharmaco-epidemiological study was to investigate whether, for GPs, there is an association between exposure to high precariousness prevalence and the subsequent inequalities in drug prescriptions between precarious and non-precarious populations. For this, using a DDD-based methodological approach and linear regression analyses, we focused on 20 selected molecules prescribed by GPs in four French regions with different precariousness prevalence.

Our findings demonstrate that exposure to high precariousness prevalence, compared to low precariousness prevalence, has a more negative and severe impact on GPs’ drug prescriptions. Contradicting our working hypothesis, this effect was observed for both precarious and non-precarious populations. Although the over-prescription of *Serenoa repens*, tamsulosine, and timolol to precarious populations is difficult to interpret, the over-prescription of rivaroxaban, rosuvastatin, tiotropium, and pyostacine, that is, drugs with easier galenic forms, was probably the result of GPs aiming to counterbalance the poor treatment compliance of precarious patients. In contrast, the under-prescription of econazole and cromolyn sodium to precarious patients, but metformin and atorvastatin to non-precarious patients suggests medical neglect, seemingly due to empathy break-down, emotional exhaustion, and associated burnout onset in GPs. Thus, we here demonstrate the detrimental impact of constant exposure to high precariousness prevalence on the quality of care.

Furthermore, the present study firstly highlights the urgency to carry out new epidemiological studies on large sample sizes to more thoroughly determine the association between empathy breakdown, burnout onset, and exposure to high precariousness prevalence in GPs. Such studies would help to detect the first signs (red flags) of empathy breakdown and burnout in GPs who are exposed to high precariousness prevalence and, therefore, prevent the deterioration of medical care. Our findings also point to the necessity of developing new training and teaching programs for medical students and experienced physicians in continuous professional education. These would aim to ameliorate the clinical practice of GPs when exposed to precarious patients, that is, firstly, to better recognize the medical characteristics of precarious populations and adapt their medical competencies to these clinical specificities; secondly, to preserve their clinical empathy and develop their capacity to self-diagnose burnout.

## Figures and Tables

**Table 1:** Mean DDD for each medication in each precarious and non-precarious population group and in each region.

Class	Medication	DDD NP (€) Mean ± SD	DDD P (€) Mean ± SD	Region	DDD NP (€) Mean ± SD	DDD P (€) Mean ± SD	DDD NP vs. DDD P (Pairwise Comparisons—One-Way ANOVA Kruskal–Wallis) *p*-Value
**A**	Metformin	6.38^−3^ ± 2.51^−1^	6.09^−3^ ± 2.35^−2^	BR	6.38^−3^ ± 2.01^−2^	6.50^−2^ ± 2.02^−1^	<0.001
				CR	9.85^−3^ ± 3.23^−2^	1.15^−1^ ± 3.81^−1^	<0.001
				OCR	9.96^−3^ ± 2.87^−2^	9.30^−2^ ± 2.71^−1^	<0.001
				OSR	4.79^−6^ ± 3.02^−7^	8.59^−7^ ± 6.78^−8^	<0.001
	Insulin glargine	2.82^−2^ ± 7.66^−2^	1.75^−3^ ± 4.68^−3^	BR	2.59^−2^ ± 4.78^−2^	2.01^−3^ ± 4.78^−3^	0.857
				CR	4.65^−2^ ± 1.11^−1^	2.66^−3^ ± 6.40^−3^	0.698
				OCR	3.47^−2^ ± 8.30^−2^	1.98^−3^ ± 4.73^−3^	0.848
				OSR	7.44^−7^ ± 2.62^−7^	4.25^−6^ ± 1.49^−6^	0.001
**B**	Acetylsalicylic acid	1.02^−0^ ± 2.11^−0^	7.08^−2^ ± 1.40^−1^	BR	1.10^−1^ ± 2.11^−0^	7.85^−2^ ± 1.44^−1^	0.883
				CR	1.38^0^ ± 2.59^−0^	8.68^−2^ ± 1.54^−1^	0.841
				OCR	1.15^−0^ ± 2.19^−0^	6.19^−1^ ± 1.62^−0^	0.822
				OSR	2.78^−6^ ± 3.95^−5^	1.51^−5^ ± 2.15^−5^	0.230
	Rivaroxaban	4.95^−2^ ± 1.44^−1^	7.71^−2^ ± 3.74^−1^	BR	6.38^−2^ ± 1.70^−1^	6.46^−3^ ± 1.93^−2^	0.671
				CR	6.36^−2^ ± 1.57^−1^	6.20^−3^ ± 1.75^−2^	0.472
				OCR	7.05^−2^ ± 1.72^−1^	6.47^−3^ ± 1.73^−2^	0.600
				OSR	1.08^−7^ ± 6.51^−7^	2.89^−1^ ± 7.21^−1^	0.008
**C**	Atorvastatin	3.54^−2^ ± 1.16^−1^	4.13^−3^ ± 1.48^−2^	BR	4.43^−2^ ± 1.24^−1^	5.36^−3^ ± 160^−2^	<0.001
				CR	5.55^−2^ ± 1.53^−1^	6.04^−3^ ± 1.87^−2^	<0.001
				OCR	4.19^−2^ ± 1.19^−1^	5.10^−3^ ± 1.57^−0^	<0.001
				OSR	9.38^−7^ ± 1.30^−7^	4.84^−6^ ± 2,37^−6^	<0.001
	Rosuvastatin	7.02^−1^ ± 1.24^−0^	1.23^−0^ ± 3.94^−0^	BR	8.28^−1^ ± 1.22^−0^	9.13^−2^ ± 1.35^−1^	0.400
				CR	1.13^−0^ ± 1.63^−0^	1.08-^−1^ ± 1.56^−1^	0.373
				OCR	8.51^−1^ ± 1.27 ^0^	1.00^−1^ ± 1.58^−1^	0.400
				OSR	7.89^−6^ ± 3.46^−6^	4.64^−0^ ± 6.98^_0^	0.001
**D**	Ciclopirox	1.06^−3^ ± 6.85^−3^	2.35^−3^ ± 2.76^−3^	BR	2.63^−3^ ± 4.80^−3^	5.77^−4^ ± 8.86^−4^	0.035
				CR	6.77^−4^ ± 1.01^−3^	3.60^−3^ ± 6.34^−3^	0.044
				OCR	9.24^−4^ ± 1.82^−3^	5.25^−3^ ± 1.15^−3^	0.056
				OSR	2.22^−6^ ± 6.29^−7^	7.86^−6^ ± 2.49^−6^	<0.001
	Econazole	2.30^−4^ ± 5.61^−4^	1.07^−3^ ± 2.69^−3^	BR	2.80^−4^ ± 5.75^−4^	1.40^−3^ ± 3.05^−3^	0.095
				CR	2.75^−4^ ± 5.37^−4^	1.21^−3^ ± 2.38^−3^	0.104
				OCR	3.63^−4^ ± 7.59^−4^	1.65^−3^ ± 3.57^−3^	0.028
				OSR	1.60^−6^ ± 9.31^−7^	7.44^−6^ ± 4.00^−6^	<0.001
**G**	Serenoa repens	1.03^−2^ ± 4.00^−2^	1.19^−2^ ± 9.93^−1^	BR	8.79^−3^ ± 2.89^−24^	4.13^−3^ ± 1.31^−2^	0.008
				CR	1.45^−2^ ± 4.68^−2^	8,90^−3^ ± 3.04^−2^	0.407
				OCR	1.79^−2^ ± 5.74^−2^	1.20^−2^ ± 3.91^−32^	0.915
				OSR	2.60^−6^ ± 2.75^−6^	4,50^−1^ ± 1.96^−0^	0.002
	Tamsulosine	1.08^−2^ ± 2.19^−2^	3.47^−2^ ± 1.39^−1^	BR	1.12^−2^ ± 1.78^−2^	5.42^−3^ ± 8.97^−3^	0.513
				CR	1.68^−2^ ± 2.79^−2^	8.22^−3^ ± 1.62^−2^	0.216
				OCR	1.50^−2^ ± 2.60^−2^	8.34^−3^ ± 1.57^−2^	0.450
				OSR	9.78^−6^ ± 6.69^−6^	1.17^−1^ ± 2.62^−1^	<0.001
**H**	Prednisolone	2.06^−2^ ± 5.44^−2^	2.09^−2^ ± 5.54^−2^	BR	2.69^−2^ ± 6.12^−2^	3.07^−3^ ± 6.53^−2^	1.000
				CR	2.22^−2^ ± 4.45^−2^	1.90^−2^ ± 3.78^−2^	1.000
				OCR	3.34^−2^ ± 7.48^−2^	3.40^−3^ ± 7.75^−2^	1.000
				OSR	1.04^−6^ ± 4.22^−7^	4.77^−6^ ± 1.96^−6^	<0.001
**J**	Amoxicillin	3.08^−3^ ± 1.01^−2^	4.72^−2^ ± 1.38^−2^	BR	4.27^−2^ ± 1.18^−2^	6.85^−3^ ± 1.62^−2^	0.593
				CR	4.43^−2^ ± 1.22^−2^	6.53^−2^ ± 1.62^−2^	0.962
				OCR	3.60^−2^ ± 1.03^−2^	5.49^−2^ ± 1.42^−2^	0.719
				OSR	9.93^−7^ ± 5.20^−7^	4.61^−6^ ± 2.47^−6^	<0.001
	Pyostacine	5.24^−2^ ± 1.00^−2^	2.11^−1^ ± 5.97^−1^	BR	6.45^−2^ ± 1.14^−1^	6.22^−2^ ± 1.08^−1^	1.000
				CR	7.30^−2^ ± 1.28^−1^	5.41^−2^ ± 9.39^−2^	1.000
				OCR	7.21^−2^ ± 1.26^−1^	5.78^−2^ ± 1.01^−1^	1.000
				OSR	9.36^−7^ ± 3.29^−7^	6.70^−6^ ± 1.17^−0^	0.273
**M**	Ibuprofen	1.04^−2^ ± 3.60^−2^	1.52^−2^ ± 3.60^−2^	BR	1.03^−2^ ± 2.86^−2^	1.66^−2^ ± 4.50^−2^	1.000
				CR	1.49^−2^ ± 4.20^−2^	1.90^−2^ ± 5.49^−2^	1.000
				OCR	1.64^−2^ ± 4.98^−2^	2.51^−2^ ± 8.03^−2^	1.000
				OSR	9.55^−7^ ± 5.24^−7^	4.43^−6^ ± 2.48^−6^	<0.001
**N**	Paracetamol	4.68^−2^ ± 2.23^−1^	3.50^−2^ ± 2.00^−1^	BR	5.98^−2^ ± 2.20^−1^	4.14^−2^ ± 1.69^−1^	0.893
				CR	7.09^−2^ ± 2.79^−1^	5.13^−2^ ± 2.49^−1^	0.845
				OCR	5.66^−2^ ± 2.64^−1^	4.73^−2^ ± 2.62^−1^	0918
				OSR	1.01^−6^ ± 4.48^−6^	4.85^−6^ ± 2.21^−6^	<0.001
**P**	Ivermectin	3.48^−3^ ± 8.45^−3^	1.60^−2^ ± 3.11^−2^	BR	6.95^−3^ ± 1.21^−2^	2.54^−2^ ± 4.46^−2^	1.000
				CR	6.98^−3^ ± 1.21^−2^	2.19^−2^ ± 3.85^−2^	1.000
				OCR	1.26^−6^ ± 1.36^−8^	1.67^−2^ ± 2.91^−2^	1.000
				OSR	6.34^−6^ ± 7.29^−7^	1.26^−6^ ± 1.36^−8^	0.281
**R**	Salbutamol	1.55^−1^ ± 7.08^−1^	3.28^−1^ ± 1.20^−0^	BR	3.53^−1^ ± 1.43^−0^	2.09^−1^ ± 8.32^−1^	0.999
				CR	3.03^−1^ ± 1.21^−0^	2.35^−1^ ± 9.27^−1^	0.910
				OCR	2.63^−1^ ± 1.00^−0^	1.76^−1^ ± 6.75^−1^	1.000
				OSR	3.92^−1^ ± 1.15^−0^	1.59^−5^ ± 1.94^−5^	<0.001
	Tiotropium	2.39^−1^ ± 3.26^−1^	3.53^−1^ ± 7.00^−1^	BR	3.39^−1^ ± 4.08^−1^	1.32^−1^ ± 1.50^−1^	0.980
				CR	3.04^−1^ ± 3.43^−1^	9.32^−1^ ± 1.00^−1^	0.750
				OCR	3.14^−1^ ± 3.36^−1^	9.15^−1^ ± 8.77^−2^	0.536
				OSR	3.62^−6^ ± 5.09^−9^	1.09^−0^ ± 1.15^−0^	0.068
**S**	Cromolyn sodium	1.11^−3^ ± 2.27^−3^	6.33^−3^ ± 1.37^−2^	BR	1.12^−3^ ± 2.05^−2^	7.25^−3^ ± 1.48^−2^	0.857
				CR	1.68^−2^ ± 2.91^−2^	9.18^−3^ ± 1.76^−2^	0.687
				OCR	1.61^−3^ ± 2.56^−2^	7.74^−3^ ± 1.32^−2^	0.848
				OSR	2.10^−6^ ± 8.98^−7^	6.94^−6^ ± 3.43^−6^	0.001
	Timolol	1.68^−3^ ± 5.57^−3^	8.90^−2^ ± 3.80^−1^	BR	1.82^−2^ ± 5.53^−2^	4.50^−2^ ± 1.47^−2^	<0.001
				CR	2.29^−2^ ± 6.19^−2^	7.87^−3^ ± 2.23^−2^	0.004
				OCR	2.60^−2^ ± 7.25^−2^	7.94^−3^ ± 2.27^−2^	0.009
				OSR	1.54^−5^ ± 1.11^−5^	3.36^−1^ ± 7.07^−1^	<0.001

Note: Mean DDD of each tested medication is shown for each precarious and non-precarious population for each region separately. Results of pair-wise comparisons between groups for each region (one-way ANOVA Kruskal–Wallis) are also shown. BR = Brittany region; CR = Center region; DDD = defined daily dose; P = precarious; NP = non-precarious; OCR = Occitany region; OSR = Overseas region; SD = standard deviation; ATC Classification (first level): A = Alimentary tract and metabolism; B = Blood and blood forming organs; C = Cardiovascular system; D = Dermatological; H = Systemic hormonal preparations, excluding sex hormones and insulins; J = Anti-infective for systemic use; M = Musculo-skeletal system; N = Nervous system; P = Antiparasitic products, insecticides, and repellents; R = Respiratory system; S = Sensory organs.

**Table 2:** Sample size and achieved power computations for each tested medication.

Medications	Sample Size				N	Achieved Power
	Non-Centrality Parameters δ	Critical *t*	Df	Min. Sample Size		
Metformin	2.5084447	1.6568452	128	132	2760	1.0000000
Insulin glargine	2.5426355	1.6802300	44	48	80	0.8998699
Acetysalicylic acid	2.5530778	1.6895725	35	39	112	0.9960272
Rivaroxaban	2.54000013	1.6735649	54	58	144	0.9903044
Atorvastatin	2.5081065	1.6623540	88	92	3128	1.0000000
Rosuvastatin	5.0575634	2.9199856	2	6	120	1.0000000
Econazole	2.5281521	1.6706489	60	64	624	1.0000000
Ciclopirox	2.50300004	1.6536580	174	178	240	0.8250026
*Serenoa repens*	2.9247437	2.0150484	5	9	404	1.0000000
Tamsulosine	2.5345654	1.6838510	40	44	512	1.0000000
Prednisolone	2.5034009	1.6567516	129	133	520	0.9983722
Amoxicillin	3.4858480	2.1318468	4	8	2136	1.0000000
Pyostacine	2.6321916	1.7291328	19	23	32	0.8500695
Ibuprofen	2.4983873	1.6515642	228	232	976	0.9992094
Paracetamol	2.4913307	1.6481729	460	464	2120	0.9996143
Ivermectin	2.5241591	1.6665997	71	75	32	0.3567551
Salbutamol	2.4919857	1.6479629	491	495	352	0.5539792
Tiotropium	2.6147066	1.7396067	17	21	48	0.9716385
Cromolyn Sodium	3.4211741	2.1318468	4	8	216	1.0000000
Timolol	2.5712845	1.6923603	33	37	560	1.0000000

The sample size (i.e., non-centrality parameters δ; critical *t*; Df; minimal sample size), sample number, and achieved power are shown for each tested medication (α err. prob. = 0.005; power (1-β err. prob.) = 0.8; number of predictors = 3). The achieved powers of ivermectin and salbutamol were found to be <0.8. These two medications were removed from the statistical testing (linear regression analyses) (note: Df = degree of freedom; N = sample number).

**Table 3:** Results of the linear regression analyses computed for each medication.

Class	Medication	Predictor	Estimate	SE	*p*-Value	Pattern
**A**	Metformin	intercept	0.1057	0.00730	<0.001	
		prevalence	−0.3198	0.04229	<0.001 *	
		population P–NP	−0.0957	0.01033	<0.001 *	
		prevalence * population	0.2896	0.05981	<0.001 *	pattern-3
	Insulin glargine	intercept	0.0441	0.0128	<0.001	
		prevalence	−0.1324	0.0740	0.077	
		population P–NP	−0.0413	0.0181	0.025*	
		prevalence * population	0.1240	0.1046	0.240	
**B**	Acetylsalicylic acid	intercept	1.51	0.286	<0.001	
		prevalence	−4.59	1.654	0.006 *	
		population P–NP	−1.41	0.404	<0.001 *	
		prevalence * population	4.28	2.339	0.070	
	Rivaroxaban	intercept	0.0820	0.0490	0.096	
		prevalence	−0.2485	0.2839	0.0383 *	
		population P–NP	−0.1462	0.0693	0.037 *	
		prevalence * population	1.3269	04014	0.001 *	pattern-1
**C**	Atorvastatin	intercept	0.0591	0.00317	<0.001	
		prevalence	−0.1811	0.01835	<0.001 *	
		population P–NP	−0.0522	0.00448	<0.001 *	
		prevalence * population	0.1600	0.02597	<0.001 *	pattern-3
	Rosuvastatin	intercept	1.17	0.510	0.024	
		prevalence	−3.56	2.953	0.230	
		population P–NP	−2.20	0.721	0.003 *	
		prevalence * population	20.86	4.177	<0.001 *	pattern-1
**D**	Ciclopirox	intercept	0.00186	0.000723	0.011	
		prevalence	−0.00610	0.00419	0.146	
		population P–NP	0.00179	0.00102	0.081	
		prevalence * population	−0.00380	0.00592	0.522	
	Econazole	intercept	0.000723	0.000165	0.023	
		prevalence	−0.00112	0.000956	0.241	
		population P–NP	0.00138	0.000234	<0.001 *	
		prevalence * population	−0.00414	0.00135	0.002 *	pattern-2
**G**	*Serenoa repens*	intercept	0.0066	0.0754	0.826	
		prevalence	−0.0483	0.4366	0.912	
		population P–NP	−0.1188	0.1061	0.263	
		prevalence * population	1.7352	0.6145	0.005 *	pattern-1
	Tamsulosin	intercept	0.0177	0.00900	0.050	
		prevalence	−0.0530	0.05210	0.309	
		population P–NP	−0.0379	0.01272	0.003 *	
		prevalence * population	0.4724	0.07368	<0.001 *	pattern-1
**H**	Prednisolone	intercept	0.03395	0.00512	<0.001	
		prevalence	−0.10179	0.02962	<0.001 *	
		population P–NP	0.000729	0.00723	0.920	
		prevalence * population	−0.00303	0.04190	0.942	
**J**	Amoxicillin	intercept	0.00516	0.000556	<0.001	
		prevalence	−0.01592	0.00322	<0.001 *	
		population P–NP	0.00278	0.000786	<0.001 *	
		prevalence * population	−0.00863	0.00455	0.058	
	Pyostacine	intercept	0.0869	0.152	0.571	
		prevalence	−0.2631	0.878	0.767	
		population P–NP	−0.1813	0.214	0.405	
		prevalence * population	2.5956	1.242	0.046 *	pattern-1
**M**	Ibuprofen	intercept	0.01701	0.00316	<0.001	
		prevalence	−0.05031	0.01832	0.006 *	
		population P–NP	0.00774	0.00447	0.084	
		prevalence * population	−0.02294	0.02591	0.376	
**N**	Paracetamol	intercept	0.0782	0.00993	<0.001	
		prevalence	−0.2395	0.05749	<0.001 *	
		population P–NP	−0.0202	0.01404	0.150	
		prevalence * population	0.0641	0.08130	0.430	
**R**	Tiotropium	intercept	0.400	0.142	0.007	
		prevalence	−1.226	0.820	0.142	
		population P–NP	−0.538	0.200	0.010 *	
		prevalence * population	4.977	1.160	<0.001 *	pattern-1
**S**	Cromolyn sodium	intercept	0.00181	0.00142	0.203	
		prevalence	−0.00541	0.00822	0.512	
		population P–NP	0.00824	0.00202	<0.001 *	
		prevalence * population	−0.02503	0.01198	0.038 *	pattern-2
	Timolol	intercept	0.0275	0.0232	0.235	
		prevalence	−0.0819	0.01342	0.541	
		population P–NP	−0.1030	0.0328	0.002 *	
		prevalence * population	1.3374	0.1898	<0.001 *	pattern-1

The effects of precariousness, population, and interaction between prevalence and population on GPs’ drug prescriptions are shown for each tested medication. The interaction pattern (prevalence * population) is also reported in the last column for each significant interaction (Note: P = precarious; NP = non-precarious; SE = standard error; * indicates a significant *p*-value).

## Data Availability

The data that support the findings of this study are available from the “Institut Statistique des Professionnels de Santé Libéraux” (ISPL), but restrictions apply to the availability of these data, which were used under license for the current study, and so are not publicly available. Data are, however, available from the authors upon reasonable request and with permission of the ISPL.

## Appendix B

**Table A1:** Results of assumption verifications computed prior to linear regression analyses.

	Normality Test Shapiro–Wilk *p*-Value	Heteroskedasticity Test Goldfeld–Quandt *p*-Value	Durbin–Watson for Autocorrelation *p*-Value	Collinearity Statistics VIF		
				Prev.	Pop.	Prev. * Pop.
Metformin	<0.001	1.000	<0.001	2.00	2.36	3.36
Insulin glargine	<0.001	1.000	0.468	2.13	2.34	3.56
Acetysalicylic acid	<0.001	0.999	0.272	2.00	2.36	3.36
Rivaroxaban	<0.001	<0.001	0.052	2.00	2.36	3.36
Atorvastatin	<0.001	1.000	<0.001	2.00	2.36	3.36
Rosuvastatin	<0.001	0.021	0.008	2.00	2.42	3.42
Econazole	<0.001	0.918	<0.001	2.00	2.36	3.36
Ciclopirox	<0.001	<0.001	0.018	2.00	2.36	3.36
*Serenoa repens*	0.018	0.104	<0.001	2.25	2.62	3.37
Tamsulosin	<0.001	<0.001	0.044	2.00	2.36	3.36
Prednisolone	<0.001	0.452	0.604	2.00	2.36	3.36
Amoxicillin	<0.001	1.000	<0.001	2.02	2.36	3.34
Pyostacine	<0.001	<0.001	0.088	2.00	2.36	3.36
Ibuprofen	<0.001	0.042	0.134	2.00	2.36	3.36
Paracetamol	<0.001	1.000	0.016	2.00	2.36	3.36
Ivermectin	<0.001	0.992	0.146	2.00	2.36	3.36
Salbutamol	<0.001	1.000	0.480	2.00	2.36	3.36
Tiotropium	<0.001	<0.001	0.848	2.00	2.36	3.36
Cromolyn Sodium	<0.001	0.940	<0.001	2.17	2.37	3.63
Timolol	<0.001	<0.001	0.002	2.00	2.36	3.36

Results of assumption verifications are shown for each tested medication (i.e., normality test (Shapiro–Wilk), heteroscedasticity test (Goldfeld–Quandt), autocorrelation test (Durbin–Watson), collinearity test (VIF)).
